# From decimeter-scale elevated ionic conductivity regions in the cloud to lightning initiation

**DOI:** 10.1038/s41598-021-97321-4

**Published:** 2021-09-09

**Authors:** D. I. Iudin, V. A. Rakov, A. A. Syssoev, A. A. Bulatov, M. Hayakawa

**Affiliations:** 1 Geophysical Electrodynamics Department, Federal Research Center Institute of Applied Physics of the Russian Academy of Sciences, 603950 Nizhny Novgorod, Russia; 2 Department of Medical Physics and Informatics, Privolzhsky Research Medical University, 603005 Nizhny Novgorod, Russia; 3 Joint Department of Space Physics with the Space Research Institute (RAS), National Research University Higher School of Economics, 101000 Moscow, Russia; 4 Department of Electrical and Computer Engineering, University of Florida, Gainesville, FL 32611 USA; 5 Laboratory of Nonlinear Physics of Natural Processes, Federal Research Center Institute of Applied Physics of the Russian Academy of Sciences, 603950 Nizhny Novgorod, Russia; 6 Advanced Wireless & Communication Research Center, The University of Electro-Communications, Chofu, Tokyo 182-8585 Japan

**Keywords:** Natural hazards, Complex networks, Nonlinear phenomena, Phase transitions and critical phenomena, Atmospheric dynamics

## Abstract

In this work, we represent the lightning initiation scenario as a sequence of two transitions of discharge activity to progressively larger spatial scales: the first one is from small-scale avalanches to intermediate-scale streamers; and the second one is from streamers to the lightning seed. We postulate the existence of ion production centers in the cloud, whose occurrence is caused by electric field bursts accompanying hydrometeor collisions (or near collisions) in the turbulent thundercloud environment. When a new ion production center is created inside (fully or partially) the residual ion spot left behind by a previously established center, there is a cumulative effect in the increasing of ion concentration. As a result, the essentially non-conducting thundercloud becomes seeded by elevated ion-conductivity regions (EICRs) with spatial extent of 0.1–1 m and a lifetime of 1–10 s. The electric field on the surface of an EICR (due to its conductivity being at least 4 orders of magnitude higher than ambient) is a factor of 3 or more higher than ambient. For a maximum ambient electric field of 100 kV/m typically measured in thunderclouds, such field enhancement is sufficient for initiation of positive streamers and their propagation over distances of the order of decimeters, and this will be happening naturally, without any external agents (e.g., superenergetic cosmic ray particles) or extraordinary in-cloud conditions, such as very high potential differences or very large hydrometeors. Provided that each EICR generates at least one streamer during its lifetime, the streamers will form a 3D network, some parts of which will contain hot channel segments created via the cumulative heating and/or thermal-ionizational instability. These hot channel segments will polarize, interact with each other, and cluster, forming longer conducting structures in the cloud. When the ambient potential difference bridged by such a conducting structure exceeds 3 MV, we assume that the lightning seed, capable of self-sustained bidirectional extension, is formed.

## Introduction

This study is an extension of work by Iudin et al.^1^ who proposed a scenario in which an essentially non-conducting thundercloud becomes seeded by decimeter-scale, long-lived elevated ionic conductivity regions (EICRs). Our review of previous works on lightning initiation is found in the Introduction section of Iudin et al.^1^. There is one more work on the subject recently published by Kostinskiy et al.^2^. In that work, the authors explained how fast positive breakdown (FPB) events, first reported by Rison et al.^3^, are produced by cosmic ray showers interacting with the so-called air electrodes (small cloud volumes with electric field exceeding the conventional breakdown threshold, created by turbulence). In contrast, in our scenario there is no need to invoke an external triggering agent (cosmic ray shower).

Formation of EICRs includes a chain of processes which is initiated by electron avalanches facilitated by highly-localized electric field amplifications in the space between colliding or nearly colliding hydrometeors. They result in the formation of ion production centers (IPCs) which have dimensions of the order of $10^{-3}{-}10^{-2}$ m and lifetimes of the order of $10^{-4}{-}10^{-3}$ s. IPCs transition to residual ion concentration spots (RICSs) with dimensions and lifetimes of the order of $0.1{-}1$ m and $1{-}10$ s, respectively. Some new IPCs will occur in a virgin air, while others will fully or partially overlap RICSs. Such overlapping provides cumulative effect in the growth of ion concentration, which is enhanced by the detachment of electrons previously attached to neutral molecules (primarily oxygen). This electron release is made possible by electric field bursts creating new IPCs. It is clear that if the rate of creation of IPCs is sufficiently high, the cumulative effect in the growth of ion concentration in parts of RICSs involved in the overlapping process can result in a significant ionic conductivity increase, which constitutes the formation of EICRs. The critical spatial-temporal frequency of IPC occurrence needed for EICR formation was estimated to be 0.1 $\text {m}^{-3} \text {s}^{-1}$ or so^1^. For comparison, hydrometeor collision rates in thunderclouds were estimated^4–7^ to be of the order of 10–$10^2$ $\text {m}^{-3} \text {s}^{-1}$ or even $10^2$–$10^3$ $\text {m}^{-3} \text {s}^{-1}$, based on in-situ measurements. This means that EICR generation becomes possible even if only 1 out of 100 to 1000 collisions is associated with ionization of air (electron avalanches). Characteristics of IPCs, RICSs, and EICRs are summarized in Table 1. Note that, although dimensions and lifetimes of EICRs are the same as those of RICSs ($0.1{-}1$ m and $1{-}10$ s, respectively), the expected conductivity of EICRs is 3 orders of magnitude higher.

**Table 1:** Characteristics of IPCs, RICSs, and EICRs^1^.

Process	Spatial scale^a^, m	Time scale^b^, s	Conductivity, S/m
Ion production centers (IPCs)	$\sim 10^{-3}{-}10^{-2}$ (small scale)	$10^{-4}{-}10^{-3}$	$10^{-5}{-}10^{-4}$
Residual ion concentration spots (RICSs)	$\sim 0.1{-}1$ (intermediate scale)	$1{-}10$	$10^{-13}{-}10^{-12}$
Elevated ionic conductivity regions (EICRs)	$\sim 0.1{-}1$ (intermediate scale)	$1{-}10$	$10^{-10}{-}10^{-9}$

^a^Processes, occurring on a spatial scale of $\sim 10{-}100$ m (or more) are considered here as large-scale processes.

^b^Time scale of IPCs is determined by the characteristic time of change in the configuration of hydrometeors, and the lifetime of RICSs and EICRs is determined by the characteristic lifetime of ions.

In this paper, we will (1) discuss the role of EICRs in the initiation of streamers, (2) show how streamers can interact with each other to form 3D streamer networks with embedded hot channel segments (such networks are called unusual plasma formations or UPFs, first reported by Kostinskiy et al.^8^), and (3) examine the conditions needed for creation of lightning seed that has the potential to eventually become a self-propagating lightning discharge. The preliminary breakdown process is outside the scope of this study. It is worth noting that UPFs have been also invoked in their lightning initiation theory by Kostinskiy et al.^2^. However, UPFs in their theory occur in the cloud in response to an external impact (cosmic ray shower), while in our scenario UPFs are formed due to the internal dynamics of thundercloud itself. “Methods” section contains details on our estimation of the critical rate of occurrence of streamers and a description of the model used to illustrate the formation of lightning seed.

## Proposed scenario

### Initiation of streamers

The presence of decimeter-scale EICRs in the thundercloud should facilitate the initiation of streamers that can potentially lead to creation of lightning seed. It is important to note that in our scenario we assume that streamers will form a 3D network with complex dynamics, as opposed to the onset and evolution of a single streamer originating from a hydrometeor considered in some previous studies (e.g., Sadighi et al.^9^ and Babich et al.^10^). 3D streamer formations (networks) in thunderclouds were previously considered in studies^2,3,8,11,12^ among others.

As seen in Table 1, the expected conductivity $\sigma$ of EICRs of $10^{-10}$ to $10^{-9}$ S/m significantly exceeds the background cloud conductivity of $10^{-14}$ S/m or less. The corresponding Maxwellian relaxation time for EICRs, given by $\varepsilon _0/\sigma$, where $\varepsilon _0$ is the permittivity of free space, is approximately $10^{-1}$ to $10^{-2}$ s (100 to 10 ms). As a result, EICRs, whose life time is 1–10 s, will polarize in the ambient electric field faster than they can be destroyed by recombination, losses to hydrometeors, or cloud turbulence. Note that due to the ion drift in the ambient electric field EICRs have an elongated shape oriented along the ambient electric field, with one end being dominated by positive ions and the other one by negative ions. Spatial fluctuations of volume charge density create intermediate-scale (0.1–1 m) electric field enhancements which cause a gradual increase in the rate of occurrence of new IPCs, thereby closing the positive feedback loop. Indeed, in the presence of EICRs, IPCs will be formed not only due to collisions or nearly collisions of hydrometers, but also due to interaction of hydrometeors with EICRs. The electron avalanches, which are produced by highly localized (small-scale; $10^{-3}$–$10^{-2}$ m) electric field bursts in the vicinity of colliding (or nearly colliding) hydrometeors, are thought to develop into positive streamers if the intermediate-scale electric field fluctuations exceed the critical field for positive streamer propagation $E_s^+$, often assumed to be $5\times 10^5$ V/m at ground (sea) level (e.g., Bazelyan and Raizer^13^). This value is similar to the experimentally estimated (e.g., Les Renardieres Group^14,15^) average electric field intensity (4.5 to $5\times 10^5$ V/m) in the positive streamer zone of long positive sparks and to the so-called stability field for positive laboratory streamers (e.g., Allen and Mikropoulos^16^).

At cloud altitudes, due to lower air density, $E_s^+$ is lower than at sea level. Multiplying $5\times 10^5$ V/m by the ratio of air density at a given altitude to that at sea level yields $E_s^+$ at that altitude. Specifically, $E_s^+$ is equal to $2.3\times 10^5$ V/m at an altitude of 6 km and $1.8\times 10^5$ V/m at an altitude of 8 km. Those values correspond to clear air and should be further reduced by the presence of hydrometeors. Macky^17^ observed this effect for raindrops with radii of 0.85 to 2.6 mm, but it should also occur for ice crystals which have relative electric permittivity $\varepsilon _r$ (at dc to 10 Hz or so) of the order of 100 (see, for example, Table I of Evans^18^) and sharp edges. Riming ice and graupel (millimeter-size snow pellet) particles should also contribute, because compact wet snow is characterized by $\varepsilon _r=50$ and electric conductivity of $10^{-6}$ S/m^19^. On the basis of laboratory experiments and modeling, Phelps and Griffiths^20^ and Griffiths and Phelps^11^ estimated $E^+_s$ to be $1.5\times 10^5$ V/m (150 kV/m) at an altitude of about 6.5 km. We will use it in the following discussion. Further, throughout this paper, we use electric field values corresponding to specific altitudes (along with the altitude value), as opposed to those normalized to the sea-level air density (the so-called sea-level equivalents). Note that the field threshold for streamer onset is higher than that for streamer propagation. Specifically, the field thresholds for the onset of positive streamers from various types of hydrometeors are in the range of 2.5 to $9.5\times 10^5$ V/m (250 to 950 kV/m) at cloud altitudes^21–25^.

The magnitude of spatial variation of the volume charge density of ions is limited only by ion losses to hydrometeors and ion-ion recombination. The characteristic value of charge of either polarity $Q_s$ contained in an ion spot (RICS or EICR) is approximately $Q_s\simeq 4\pi er_0^3{\bar{n}}_n/3\simeq 5\cdot 10^{-7}$ C^1^ ($e=1.6\cdot 10^{-19}$ C, $r_0\approx 2$ mm, and ${\bar{n}}_n\simeq 10^{20}$ $\text {m}^{-3}$ are the absolute value of electron charge, the linear scale of an ion production center, and the ion concentration saturation level, respectively), which is much larger than the observed maximum charge values $Q_h$ on large hydrometeors that are only $\simeq 2\cdot 10^{-10}$ C (see study^26^). Even at a distance of 10 cm from the center of ion-spot charge $Q_s$, the electric field exceeds the critical field $E_s^+\simeq 150$ kV/m for positive streamer propagation at 6.5 km altitude. Thus, the appearance in the cloud of ion spots (RICSs and EICRs) with charges at the level of $Q_s$ leads to a significant increase in the magnitude of electric field enhancements on the $\sim 0.1{-}1$ m spatial scale. As a result, the likelihood of the occurrence of streamers will increase, compared to the configuration in which only hydrometeors are taken into account.

Specifically, because the conductivity of EICRs, $10^{-10}{-}10^{-9}$ S/m, is al least 4 orders of magnitude higher than ambient, the electric field on their surface is 3 times higher than ambient, if they were spherical, or more (along the major axis), if they were ellipsoidal. For a typical maximum ambient electric field of 100 kV/m, such field enhancement on the EICR surface is likely to be sufficient for initiation of positive streamers and their propagation over distances of the order of decimeters, and this will be happening naturally, without any external agents (e.g., energetic cosmic ray particles) or extraordinary in-cloud conditions, such as very high potential differences or very large hydrometeors. Further extension is possible via the cumulative effect, when later streamers retrace the remnants of earlier ones. Griffiths and Phelps^11^, who studied initiation of streamers from hydrometeors, reported that three to seven conical streamer systems, each one passing into the debris of its predecessors, can give rise to a local field enhancement up to 1.5 MV/m over a distance of a few meters. This finding implies an increase of the conductivity of the streamer formation due to its cumulative conditioning. In our scenario, a similar overlapping of streamer paths is likely if the rate of occurrence of streamers from EICRs is sufficiently high (see “Methods” section).

### Formation of 3D streamer networks and UPFs

We assume that, due to the presence of EICRs, streamers in our scenario are not rare events; they are initiated, develop, and decay (most of them) throughout the high-field region of the cloud, where intermediate-scale electric field fluctuations exceed the field threshold for their onset and propagation. It is generally acknowledged (e.g., Nijdam et al.^27^) that in the case of multiple streamers retracing the same path, residual charges, active species, and heating associated with earlier streamers have a great impact on the characteristics of later streamers. This was demonstrated in studies of repetitively laboratory streamer discharges, where the physical and chemical reactions during the decay phase play a very important role in elevating the ionization and heating levels above the natural ones. In thunderclouds, a 3D time-varying array of EICRs serves to generate streamers, some of which can retrace the remnants of previously generated streamers. If the rate of generation of streamers is sufficiently high (which is shown to be the case in “Methods” section), an extensive spatial-temporal hierarchical system of interacting streamer channels at different stages of development with embedded hot segments (discussed below) is formed. The hot channel segments are more likely to maintain their relatively high conductivity until the occurrence of new streamers.

**Figure 1:**
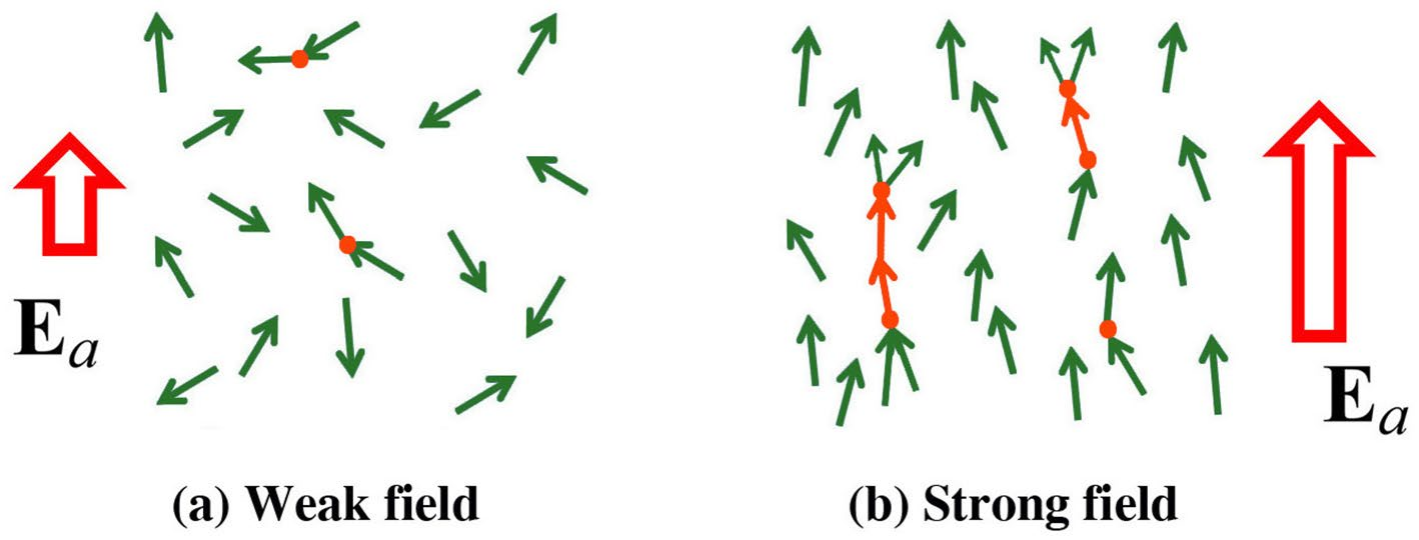
Schematic representation of the formation of volume-filling streamer network in (**a**) a relatively weak and (**b**) a relatively strong ambient electric field $E_a$. Streamers are represented by green arrows and embedded hot segments are shown by small orange circles (streamer junction points) and arrows (thermalized streamers). In (**a**), the emerging streamers can be oriented in essentially any direction, while in (**b**) streamers are predominantly oriented (and are clustering) along the ambient electric field direction.

Evolution of the volume-filling streamer network strongly depends on the magnitude of external large-scale (ambient) electric field $E_a$. In the case of weak ambient field, the emerging streamers can extend essentially in any direction (see Fig. 1a) and their length rarely exceeds the extent of intermediate-scale (0.1–1 m) fluctuations of electric field. Hot channel segments can occasionally appear (due to the so-called thermal-ionizational instability^8^) within the streamer network, but their lifetime and length are limited. As the magnitude of the ambient electric field $E_a$ increases, the influence of its direction on the direction of streamers becomes more significant (see Fig. 1b), which facilitates the interaction of streamers and their clustering. Hot channel segments can polarize and grow. Note that the embedded hot segments can grow along the ambient field, even if $E_a$ is lower than the critical field for positive streamer propagation $E_s^+$.

Maximum electric fields measured in thunderclouds with balloons are typically $(1{-}2)\times 10^5$ V/m [^28^, Table 3.2]. Maximum fields measured with aircraft and rockets are generally higher, $(3{-}4)\times 10^5$ V/m. Specifically, Winn et al.^29^ reported a peak horizontal field of the order of $4\times 10^5$ V/m extending over a distance of a few hundred meters at about 6 km altitude above sea level. Our scenario of lightning initiation does not require electric fields higher than those measured in thunderclouds.

**Figure 2:**
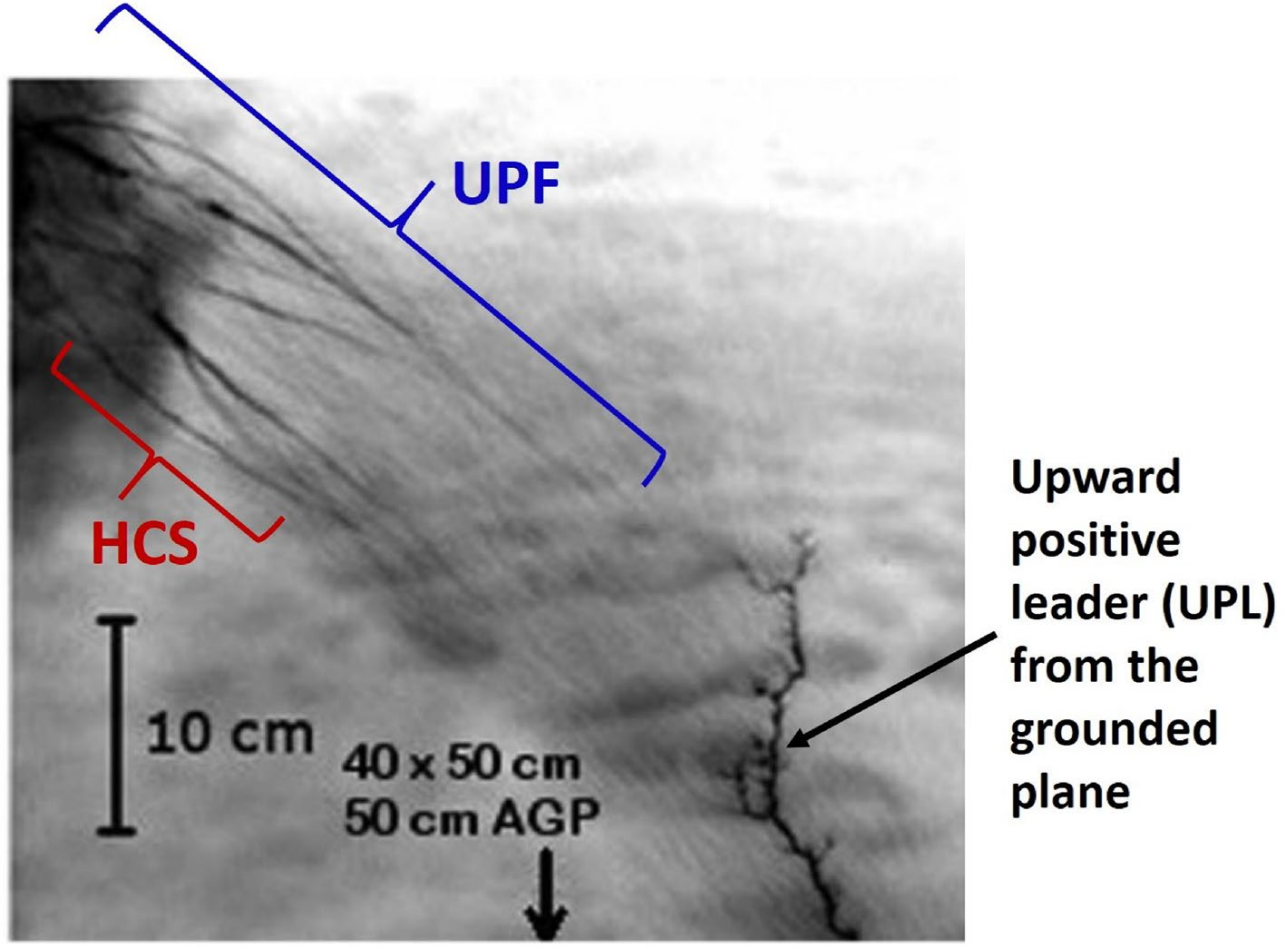
Infrared image (negative) that shows an unusual plasma formation (UPF) with embedded hot channel segments (HCS) inside the cloud of small water droplets. Also seen in this image is an upward positive leader (UPL) from the grounded plane, whose channel is expected to be hot. Note that HCS and UPL have about the same infrared brightness and, hence, about the same temperature. The entire region shown is inside the cloud. Exposure time was 6.7 ms. AGP = above the grounded plane. Adapted from [^8^, Fig. 5b].

Dwyer and Uman [^12^, section 2.2, p. 161] suggested that an extended streamer network can be created in a thundercloud, with many streamers feeding their current into a narrow channel segment where localized heating takes place. In other words, a relatively hot channel segment concentrates many partial network currents on itself and becomes hotter. This process makes the streamer network highly non-uniform in terms of its electric current and conductivity. A stochastic model of 3D streamer formations (with initial conductivity of the order of $10^{-11}$ S/m) evolving into bidirectional leaders with hot trunks was developed by Iudin et al.^30^. As of today, there is no direct (experimental) evidence of the formation of nonuniform 3D streamer networks in thunderclouds. However, such networks have been documented in artificially charged clouds of small water droplets. Specifically, Kostinskiy et al.^8^ observed the so-called unusual plasma formations (UPFs) in artificial clouds of negatively charged water droplets. The UPFs appeared as decimeter-scale streamer networks with embedded hot channel segments interacting with each other in a complex way. An example of UPF is shown in Fig. 2. Kostinskiy et al.^8^ found the hot segments of UPFs to be at about the same temperature as that of the hot leader channel observed in the same experiments and to live for milliseconds. The hot segments within UPFs are apparently formed due to the so-called thermal-ionizational instability^8^. Multiple brighter (and possibly hot) channel segments were also observed in visible-range high-speed camera recordings of negative corona streamer bursts in natural lightning (e.g., Petersen and Beasley^31^ and Qi et al.^32^). Those were attributed to space stems/leaders. Note that the temperature of a hot leader channel is about 5000 K or more, and, hence, hot channel segments should have conductivity in excess of 1 S/m (about 2 S/m at 4000 K and 30 S/m at 5000 K^33^). Kostinskiy et al.^8^ suggested that UPFs can be an intermediate stage between the initial low-conductivity streamers and a hot, self-propagating leader channel, provided that the hot segments of UPF can get polarized and grow within its overall channel network, thereby tapping electrostatic energy from a relatively large cloud volume. Kostinskiy et al.^8^ also stated that UPFs can serve to “metalize” a region of thundercloud, as described by Iudin et al.^34^, thereby creating a “seed” needed for lightning initiation. It is worth noting that parts of UPFs that are not hot (composed of streamers only) are expected to have conductivity that is much higher than ambient and, hence, serve to enhance the electric field at the periphery of UPF. Indeed, conductivity estimated for various streamer formations is of the order of $10^{-5}$ S/m^35–37^ vs. $10^{-14}$ S/m or less for the electrically undisturbed cloud environment.

As shown in “Methods” section, according to the percolation-theory-based criterion, the critical rate of occurrence of streamers leading to creation of a 3D network is 0.1 $\text {m}^{-3} \text {s}^{-1}$. Hot channel segments within the network are formed via cumulative heating and/or thermal-ionizational instability.

### Conditions needed for creation of lightning seed

**Figure 3:**
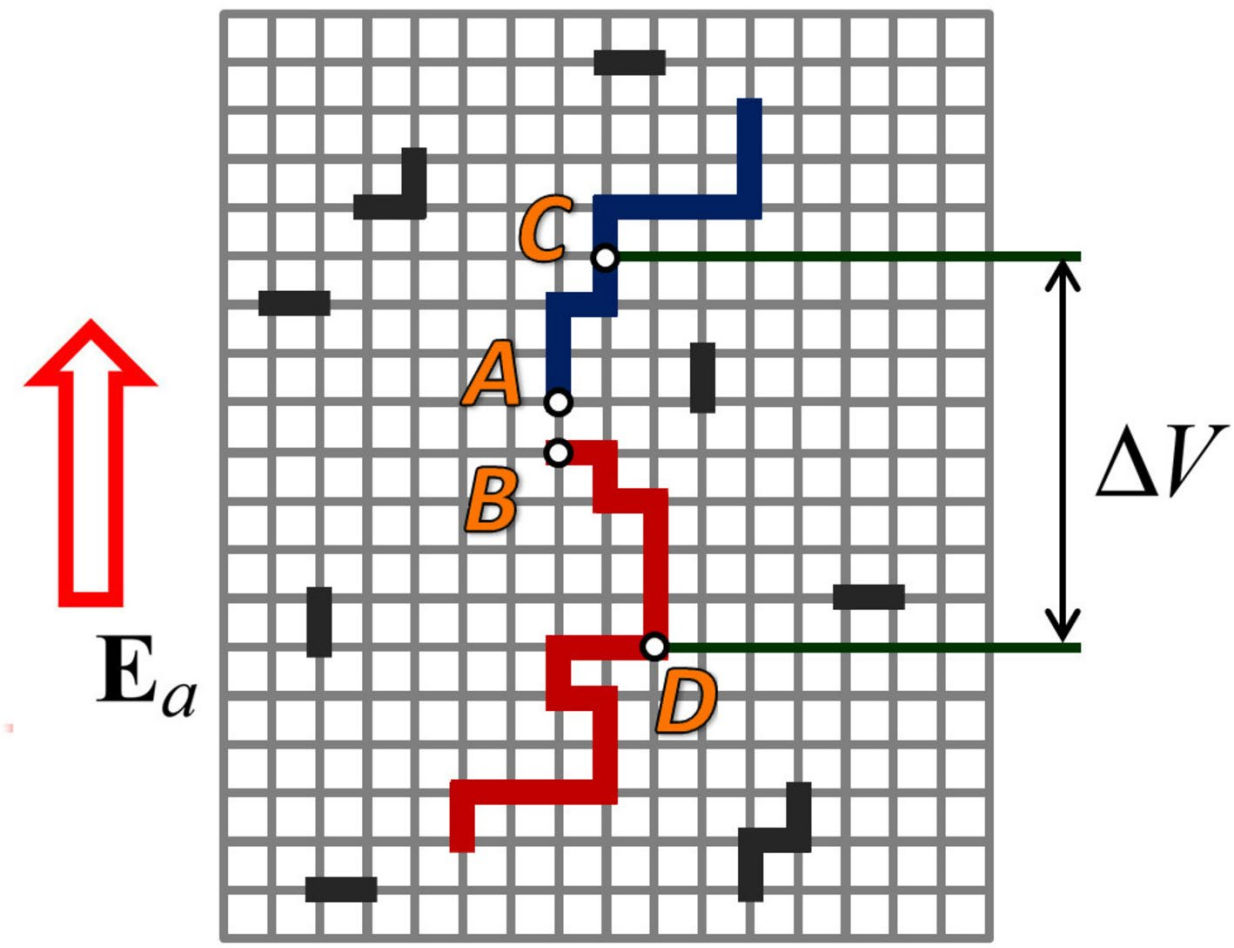
2D dielectric lattice with some conducting links and groups of interconnected conducting links (shown by thicker lines) in a uniform electric field $E_a$. The conducting links, particularly their clusters oriented primarily along $E_a$ (see the blue and red clusters whose centers (origins) are labeled C and D, respectively), can significantly enhance the electric field near cluster ends. This may lead to electrical breakdown at cluster ends (for example, and most likely, between points A and B).

In this section, we will discuss the transition from a 3D streamer network with embedded relatively small hot segments to an elongated conducting structure that is sufficiently long to polarize and extend (in a sustained manner) in the ambient electric field of the order of $10^5$ V/m. In order to illustrate the essence of this transition, we will start with a simple example of 2D rectangular lattice (see Fig. 3), whose non-conducting links are shown by thinner gray lines and conducting links or groups of interconnected links are shown by thicker black, blue, and red lines. Initially, all the links were non-conducting and any horizontal line passing through the lattice was equipotential. Then conducting links/clusters are introduced with blue and red clusters developing bidirectionally from points C and D, respectively. Potentials of points C and D are each an average of the ambient potentials at the ends of blue and red clusters, respectively.

It is well known that the presence of conductive inclusions in dielectric material dramatically reduces the external electric field that is necessary to cause electrical breakdown of the dielectric material^38^. The breakdown field is a sensitive function of the volume fraction of conductive elements embedded in the dielectric material, which is related to the formation of conductive clusters (interconnected conductive elements) in the material. The effective breakdown field was found^38^ to be inversely proportional to the length of the largest of the conductive clusters. Referring to Fig. 3, consider two largest conducting clusters oriented primarily along the uniform ambient electric field $E_a$. Let’s assume that the upper (blue) cluster whose center (origin) is labeled C is at potential $V_1$, and the lower (red) cluster with its center (origin) labeled D is at potential $V_2$. The resultant potential difference between C and D is $\Delta V=V_2-V_1$. If we assume that the blue and red clusters are perfectly conducting and, hence, each of them is equipotential, $\Delta V$ will be applied across the small gap between points *A* and *B* and will likely cause electrical breakdown in that gap (which would not have occurred in the absence of the blue and red clusters), leading to interconnection of the blue and red clusters. The likelihood of breakdown depends on the ambient electric field $E_a$ and the distance between points C and D (assumed here to be representative of the average ambient potentials corresponding to the blue and red clusters, respectively), which can be related to ambient potential differences bridged by the conducting clusters.

We now consider a conducting (hot) channel segment in the cloud, which is created via cumulative heating and/or thermal-ionizational instability. In order for a conducting segment to become capable of polarizing and self-extending (in opposite directions with respect to its origin), it is necessary, for a given ambient electric field $E_a$, that the segment attains a sufficiently large length $L=h_1+h_2$, such that:

1 $$\begin{aligned} \int _{z-h_1}^{z+h_2}({\mathbf {E}}_a\cdot {\mathbf {z}}_0)\mathrm {d}z\gtrsim \Delta V_{cr}, \end{aligned}$$

where ${\mathbf {z}}_0$ is the unit vector in the ambient field direction (assumed to be vertical), $h_1$ and $h_2$ are the vertical lengths of the segment parts below and above the origin, respectively, *z* is the coordinate of the conducting segment origin, and $\Delta V_{cr}$ is the critical ambient potential difference that allows the conducting channel segment to become self-extending. A similar criterion for initiation of upward lightning was used by Bazelyan et al. [^39^, p. 100].

We assume that $\Delta V_{cr}\approx 3$ MV. There exists some experimental evidence supporting this assumption, as discussed next. Willett et al.^40^, who used electric field sounding rockets in Florida, analyzed ambient electric fields which are capable of initiating and supporting the growth of upward leaders of the positive polarity in rocket-triggered lightning. It was established that lightning can start from the grounded triggering wires with lengths of approximately 400 m if the ambient fields aloft are about 13 kV/m. At the moment of lightning initiation, the difference between the ambient potential at the triggering-rocket altitude and that of the ground surface was − 3.6 MV. Willett et al.^40^ referred to the magnitude of this potential as triggering potential. The first measurable current pulses (precursors) in the triggering wire associated with multiple electrical breakdowns occurring near the tip of the wire that precede the initiation of self-sustaining positive leader, were detected at similar fields aloft but at wire-top heights being about 200 m or less, so that the corresponding potential was 1.3 MV. Pierce^41^, who examined lightning triggering by both grounded and ungrounded objects, found that for different conductor lengths and ambient electric fields the “potential discontinuity” (for grounded objects of height *h* it was estimated as $E_a\cdot h$, and for floating objects of length *L* as $E_a\cdot (L/2)$) was of the order of 1 MV. Specifically, for Apollo 12 (struck by lightning in 1969), whose effective length (including the exhaust trail) at the time of lightning strike to it was $\sim$ 400 m and the ambient field was $\ge$ 10 kV/m, the “potential discontinuity” was estimated to be $\ge$ 2 MV. For comparison, the left-hand side of criterion (1) for Apollo 12 would be $\ge$ 4 MV, not far from our assumed value of $\Delta V_{cr}\approx$ 3 MV. Thus, it appears that our assumed, based on the works of Willett et al.^40^ and Pierce^41^, value of $\Delta V_{cr}$ is reasonable. If condition (1) is satisfied, we assume that the lightning seed is formed.

**Figure 4:**
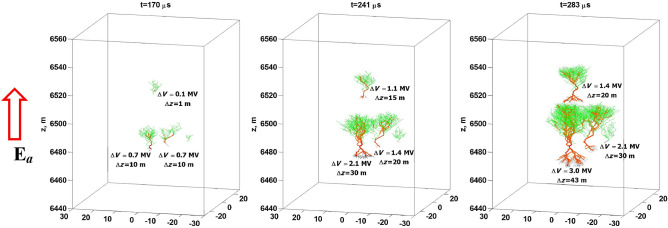
An example of the evolution (three snapshots) of 3D streamer/hot-channel systems in a uniform upward-directed electric field $E_a=70$ kV/m. Channel segments with conductivities higher than 1 S/m (assumed to be “hot”) are shown in red. Ambient potential differences $\Delta V$ bridged by hot channel segments and their vertical lengths $\Delta z$ (same as *L* in the text) are given in each panel. All three axes are labeled in meters.

Hot channel segments within the streamer network under condition ${\mathfrak {M}}_s>{\mathfrak {M}}_s^*$, i.e. when the streamer occurrence rate ${\mathfrak {M}}_s$ exceeds its critical value ${\mathfrak {M}}_s^*$ (see “Methods” section), gradually evolve into expanding conducting clusters, as illustrated in Fig. 4. In the example shown in Fig. 4, the ambient electric field was set to 70 kV/m, and the threshold conductivity to consider the channel segment “hot” was set to 1 S/m. Most of those clusters fail to become sufficiently long to satisfy condition (1), and only for one cluster, whose vertical length is 43 m (see the rightmost snapshot at $t=283$ μs in Fig. 4), $\Delta V\approx 3$ MV. According to our proposed scenario, this latter cluster (lightning seed) is capable of evolving into a full-fledged lightning discharge. It is worth noting that such definition of lightning seed is different from that previously suggested by Solomon et al.^42^: an elongated plasma patch about 10 m in length with conductivity of the order of $10^{-4}$ S/m. We are not aware of any evidence that such a plasma patch can polarize fast enough and become self-extending, although its Maxwellian relaxation time is as small as 100 ns.

## Discussion and summary

Cloud discharge activity prior to a lightning flash serves to build a complex hierarchical 3D system of interacting streamer channels at different stages of development that contains hot channel segments in its overall network-like structure, similar to recently observed unusual plasma formations (UPFs) within artificial clouds of charged water droplets^8^. Hot channel segments, created via the cumulative heating and/or thermal-ionizational instability, will polarize, interact with each other, and cluster, forming longer conducting structures in the cloud, as discussed in detail by Kostinskiy et al.^2^ in the framework of another (externally triggered) lightning initiation scenario. When a sufficiently large ambient potential difference is bridged by such a conducting structure, we assume that the lightning seed is formed.

The mechanism of lightning initiation remains a subject of intense debate (see overview given in the Introduction of the companion paper^1^ and references therein), with new observational data (particularly those acquired with modern broadband VHF interferometers) quickly making previous hypotheses incomplete (e.g., studies^3,43–47^). Dubinova et al.^48^ and Rutjes et al.^49^ argued that three conditions are required for lightning initiation: a sufficiently high ambient electric field, a sufficiently large hydrometeor, and a sufficiently large number of free electrons, with all three existing at the same time and in the same location in space. They also argued that the latter condition (sufficiently large number of free electrons) can be only satisfied by the occurrence of Cosmic Ray Shower (CRS), also known as Extensive Air Shower (EAS). It should be noted, however, that a sufficiently large number of free electrons can be a controlling factor only for sub-microsecond-scale discharges (e.g., Nijdam^50^). It is known from high-voltage experiments that for electric field (voltage) risetimes longer than 1 μs seed electrons are always available from natural sources (e.g., background cosmic rays) or via detachment from negative ions to start the discharge (e.g., [^51^, p. 133]). Detachment from ions in high electric field is usually considered to be the main source (e.g., Allen et al.^52^ and Bousiou et al.^53^). Further, although CRS can play a role in lightning initiation, the reliance on CRS (entering the cloud from above) as an activator of the growth of a fast positive breakdown (positive streamer formation) makes it difficult to explain how such growth can occur in the upward direction. Both downward and upward growing fast positive breakdowns were observed by Rison et al.^3^.

Dwyer^54^ presented a mechanism for lightning initiation by the sustained runaway breakdown (more recently called relativistic runaway electron avalanche(s) or RREA). Unlike Dubinova et al.^48^ and Rutjes et al.^49^, he did not consider CRSs or presence of hydrometeors and assumed that the seed runaway electrons for RREA are supplied by the steady background cosmic rays. It was shown that the resultant RREA with positive feedback is capable of enhancing the electric field to the point where the conventional breakdown can occur. It appears that the controlling factor in Dwyer’s model is the sufficient thickness of the high-field (exceeding the runaway breakdown threshold, about $10^5$ V/m at an altitude of 8 km) region, needed to produce substantial avalanche multiplication. This leads to a requirement of very large potential differences in the cloud. For example, for an ambient electric field of 130 kV/m at 8 km, the potential difference across the high-field region needs to be 450 MV. For comparison, Marshall and Stolzenburg^55^, from 15 balloon soundings of the electric field through thunderstorms, estimated a maximum value of the potential within a cloud relative to the Earth in the range from − 102 to + 94 MV.

There are many theoretical/modeling studies concerned with the initiation of a streamer from a single hydrometeor (e.g., studies^9,10,56^) or from an assembly of two hydrometeors (e.g., studies^57–59^) in the so-called subbreakdown fields (lower than the conventional breakdown field of the order of 1 MV/m at cloud altitudes). Significant results shedding some light on the conditions that are necessary for initiation of streamers from hydrometeors were obtained. Liu and Dwyer^60^ suggested that lightning initiation may begin with many small-scale discharges randomly occurring between oppositely charged hydrometeors in a localized thundercloud region. However, none of those studies considered the formation of hot lightning seed, capable of becoming a self-propagating discharge process, which is one of the key elements of our proposed scenario. The importance of this stage of the lightning initiation process was discussed, for example, by Rakov and De Carlo^61^.

Iudin et al.^1^ have shown how the essentially non-conducting thundercloud becomes seeded by EICRs with spatial extent of 0.1–1 m and a lifetime of 1–10 s. In the present study, assuming a realistic (of the order of 100 kV/m at 6.5 km altitude) ambient electric field, we show that the electric field on the surface of an EICR (due to its conductivity being at least 4 orders of magnitude higher than ambient) is a factor of 3 or more higher than ambient. For a maximum ambient electric field of 100 kV/m typically measured in thunderclouds, such field enhancement is sufficient for the initiation of positive streamers and their propagation over distances of the order of decimeters, and this will be happening naturally, without any external agents (e.g., superenergetic cosmic ray particles) or extraordinary in-cloud conditions, such that very high potential differences or very large hydrometeors. Provided that each EICR generates at least one streamer during its lifetime, the streamers will form a 3D network, some parts of which will contain hot channel segments created via the cumulative heating and/or thermal-ionizational instability. These hot channel segments will polarize, interact with each other, and cluster, forming longer conducting structures in the cloud. When the ambient potential difference bridged by such a conducting structure exceeds 3 MV, we assume (based on the lightning triggering potentials measured by Willett et al.^40^ and estimates made by Pierce^41^) that the lightning seed, capable of self-sustained bidirectional extension, is formed.

In the proposed scenario we acknowledge that electrons in the thunderclouds are very short-lived (attachment time is some tens of nanoseconds) and place emphasis on ions. Hydrometeors do play an important role but, unlike previous studies, only via their collisions or nearly collisions which lead to the formation of embryos of EICRs. They also help with the local electric field enhancement at the streamer-network formation stage, although decimeter-scale EICRs are more important in this process than millimeter-scale (or smaller) hydrometeors. Cosmic rays, along with the natural Earth radioactivity, enter our proposed scenario only as suppliers of seed electrons (known to be readily available, directly or via detachment, in electric fields increasing on time scales longer than 1 μs or so) for the avalanches leading to the formation of embryos of EICRs. No superenergetic particles creating cosmic ray showers are needed.

To the best of our knowledge, our proposed lightning initiation scenario not relying on an external triggering agent, which is presented by Iudin et al.^1^ and in the present paper, is the most complete one to date. However, it does contain a number of assumptions (clearly identified in the paper) that are in need of further confirmation by observations. Also, it is limited to the formation of lightning seed and does not include the preliminary breakdown and stepped leader, which are left for a future study.

Iudin et al.^1^ have demonstrated that the collective dynamics of charged hydrometeors in the thundercloud turbulent flow play a fundamental role in the redistribution and dissipation of electrostatic energy in thunderstorms. The main reservoirs for accumulating electrostatic energy in thunderclouds are (i) the large-scale electric field of the main charge regions that are formed due to the large-scale separation of oppositely charged hydrometers by gravity and updrafts, (ii) the intermediate-scale field of charged hydrometeors moving in the turbulent air flow, and (iii) the small-scale field of net and polarization charges on the surface of individual hydrometeors. The three spatial scales on which energy can be stored correspond to the three scales of energy dissipation (or three scales of discharge activity serving to relax the electric field): electron avalanches (small scale, 0.1–1 cm), streamers (intermediate scale, 0.1–1 m), and formation of lightning seed (large scale, 10–100 m). The process leading to electrostatic energy dissipation via a full-fledged lightning discharge starts with small-scale electron avalanches in the vicinity of colliding hydrometers and then proceeds to larger spatial scales, with the lightning initiation process presented here culminating in the formation of lightning seed. Since three different spatial scales are involved into the process of cloud electrostatic energy dissipation, it is convenient to represent the lightning initiation scenario as a sequence of two transitions of discharge activity to progressively larger spatial scales: the first one is from the small-scale avalanches to the intermediate-scale streamers, and the second one is from the streamers to the lightning seed. The first transition was considered by Iudin et al.^1^, who have shown how the essentially non-conducting thundercloud becomes seeded by the elevated-ionic-conductivity regions (EICRs) with spatial extent of 0.1–1 m and a lifetime of 1–10 s, and the second transition (involving the unusual plasma formations or UPFs) is presented in this paper.

## Methods

### Critical rate of occurrence of streamers

**Figure 5:**
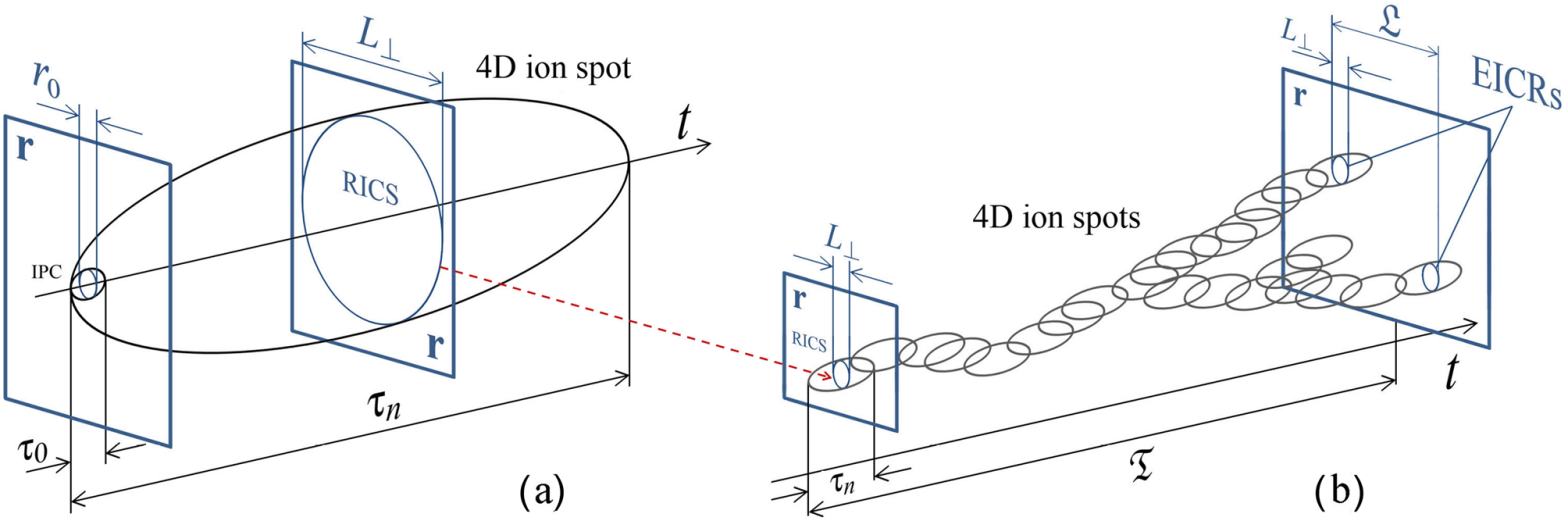
Schematic representation of (**a**) the formation of a residual ion concentration spot (RICS) and (**b**) evolution of overlapping RICSs into elevated ionic conductivity regions (EICRs).

Our proposed scenario requires an estimate of the critical rate of occurrence of streamers, which is obtained below. According to Iudin et al.^1^, collisions or nearly collisions of hydrometeors give rise to millimeter- to centimeter-scale ion production centers (IPCs). IPCs significantly increase the ionization rate, which leads to the formation of decimeter-scale residual ion concentration spots (RICSs, see Fig. 5a) and elevated ionic conductivity regions (EICRs, see Fig. 5b). EICRs are characterized by the same spatial and time scales as RICSs, but the conductivity of the former is 3 orders of magnitudes higher than that of the latter (see Table 1). An ensemble of overlapping RICSs is a complex network that is embedded in four-dimensional space-time continuum (see Fig. 5b). Due to the unidirectional property of time, the structure of this network is highly anisotropic and is characterized by time extent ${\mathfrak {T}}$ and spatial extent ${\mathfrak {L}}$, which are, respectively, parallel and perpendicular to the time axis. The critical behaviors of the time extent and the spatial extent are described by the following relationships^62^:

2 $$\begin{aligned} {\mathfrak {T}}\simeq \tau _n|{\mathfrak {B}}-{\mathfrak {B}}_c|^{-\nu _{||}} \end{aligned}$$

and

3 $$\begin{aligned} {\mathfrak {L}}\simeq L_{\perp }|{\mathfrak {B}}-{\mathfrak {B}}_c|^{-\nu _{\perp }}, \end{aligned}$$

where $\tau _n$ ($\approx 1$ s) is the RICS’s lifetime, $L_{\perp }$ ($\approx 1$ m) is the dimension of RICS, and ${\mathfrak {B}}$ is the dimensionless filling factor, with ${\mathfrak {B}}_c$ being its critical value. Spatial-temporal dimensions (2) and (3) each have a clear geometric meaning. Below the directed percolation threshold, ${\mathfrak {B}}<{\mathfrak {B}}_c$, they describe the characteristic sizes of the cluster of RICSs. Above the threshold, ${\mathfrak {B}}>{\mathfrak {B}}_c$, they give the dimensions of the characteristic spatial-temporal “holes” in the percolation network of ion spots (RICSs and EICRs). At the threshold of an exponential increase in the concentration of ions (${\mathfrak {B}}={\mathfrak {B}}_c$), this network is a directed percolation cluster: ${\mathfrak {T}}\rightarrow \infty$ and ${\mathfrak {L}}\rightarrow \infty$ or, in practice, ${\mathfrak {T}}\gg \tau _n$ and ${\mathfrak {L}}\gg L_{\perp }$.

**Figure 6:**
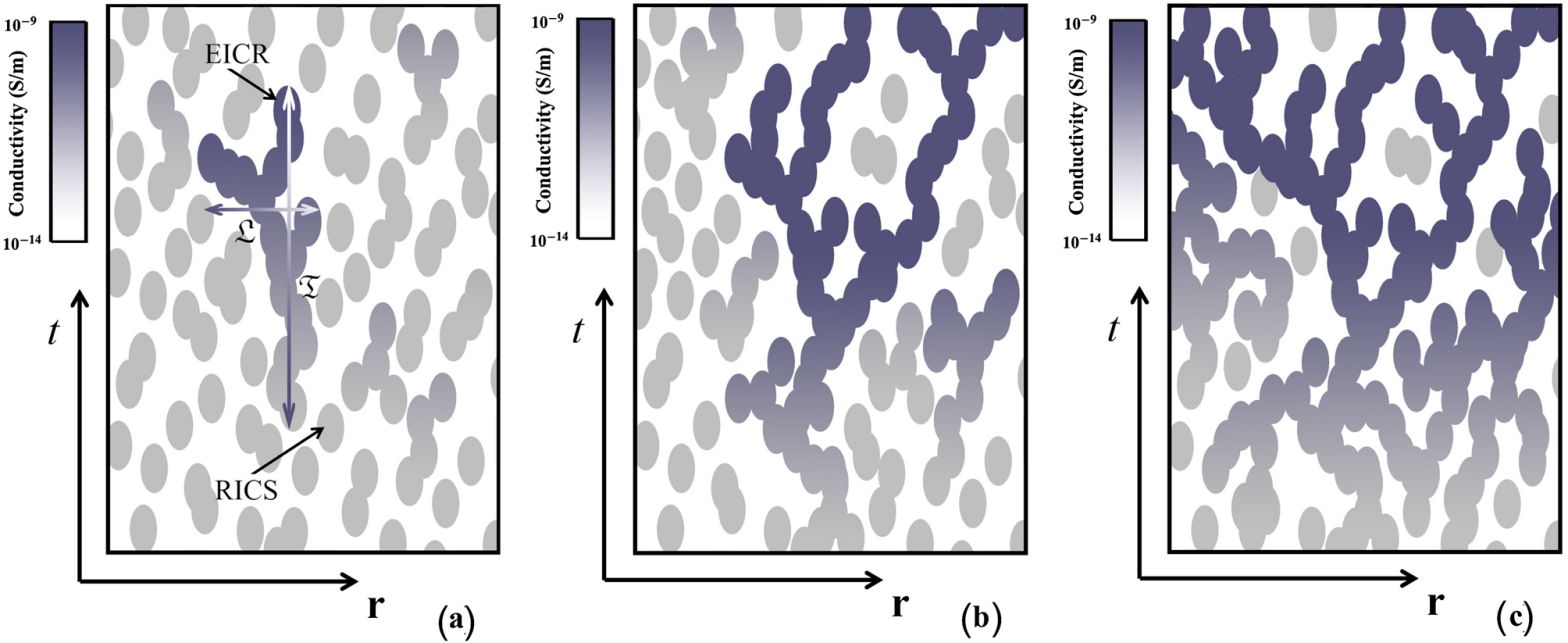
Schematic representation of overlapping RICSs that gradually lead to the formation of EICRs at different rates of occurrence of RICSs: (**a**) ${\mathfrak {M}}\lesssim {\mathfrak {M}}_c$, (**b**) ${\mathfrak {M}}={\mathfrak {M}}_c$, (**c**) ${\mathfrak {M}}\gtrsim {\mathfrak {M}}_c$. The darker the spot, the higher its conductivity.

Suppose that a chain of *k* overlapping RICSs leads to the formation of EICRs (see Fig. 5b). We assume that the first EICRs appear when RICS cluster time extent or lifetime is

4 $$\begin{aligned} {\mathfrak {T}}\simeq k\tau _n \end{aligned}$$

with $k \lesssim 10$. Using expression (2) it can be shown that (4) is satisfied when ${\mathfrak {B}}={\mathfrak {B}}^*\simeq 5\cdot 10^{-2}<{\mathfrak {B}}_c \simeq 13\cdot 10^{-2}$. When ${\mathfrak {B}}={\mathfrak {B}}^*$, the rate of EICR occurrence relative to the rate of IPC occurrence is approximately $10^{-3}$ (see Fig. 6a). Then, as ${\mathfrak {B}}$ approaches its threshold value ${\mathfrak {B}}_c$, the EICR rate relative to to the IPC rate gradually increases to a value of the order of $2\cdot 10^{-2}$ (see Fig. 6b). Above the threshold ${\mathfrak {B}}>{\mathfrak {B}}_c$, the growth of the occurrence rate of EICRs increases and reaches unity somewhere in the range ${\mathfrak {B}}_c<{\mathfrak {B}}<1$, when almost every IPC leads to the formation of EICR (see Fig. 6c). Thus, Fig. 6 shows that a slight increase in the rate of occurrence of IPCs near the threshold level causes a sharp increase in the fraction of EICRs in the total number of ion spots. Within the framework of the proposed scenario, the rate of occurrence of EICRs is of the order of 0.1 $\text {m}^{-3} \text {s}^{-1}$.

It is reasonable to assume that each EICR generates at least one streamer during its lifetime (1–10 s). Then the rate of occurrence of streamers will be no less than 0.1 $\text {m}^{-3} \text {s}^{-1}$. Using the percolation-theory-based criterion^1,62^, one can estimate the rate of streamer generation that leads to the cumulative heating and gradual increase of the conductivity of streamer channels. This approach is similar to that used by Iudin et al.^1^ in finding the critical rate of occurrence of ion production centers leading to formation of EICRs. The critical rate of occurrence of streamers is given by

5 $$\begin{aligned} {\mathfrak {M}}_s^*\simeq \displaystyle \frac{{\mathfrak {V}}_c}{{\mathfrak {S}}_s^{\mathrm {max}}}, \end{aligned}$$

where ${\mathfrak {V}}_c\simeq 0.13$ is the threshold value of dimensionless filling factor for 4D space-time domain, and ${\mathfrak {S}}_s^{\mathrm {max}}$ is the maximum 4D volume filled with streamers at different stages of their development or decay. This volume can be estimated as

6 $$\begin{aligned} {\mathfrak {S}}_s^{\mathrm {max}}=L^3\cdot T_m^{\mathrm {max}}, \end{aligned}$$

where *L* is the intermediate scale of electric field fluctuations and $T_m$ is the maximum depth of the environment (preionized air) memory. Assuming that $T_m^{\mathrm {max}}\simeq 1$ s and $L\simeq 1$ m, we obtain ${\mathfrak {S}}_s^{\mathrm {max}}\simeq L^3\cdot T_m^{\mathrm {max}}\simeq 1$ $\text {m}^{3}$s and ${\mathfrak {M}}_s^*\simeq 0.1\;\text{ m}^{-3}\text{ s}^{-1}$, which is equal to the expected rate of generation of streamers by EICRs (see above).

In summary, provided that each EICR generates at least one streamer during its lifetime, the streamers will form a 3D network, some parts of which will contain hot channel segments created via cumulative heating and/or thermal-ionizational instability.

### Model used to illustrate the formation of lightning seed

Here we describe the model that was used to produce the results presented in Fig. 4. The approach is, in general, similar to that employed in the studies of Iudin et al.^30^ and Syssoev et al.^63,64^, except for several features, which are described below.

It is assumed that the computational domain, which is a rectangular box, divided into $60\times 60\times 120$ cubical cells with an edge of 1 m, is located at an altitude of 6.5 km above sea level. An upward-directed ambient electric field is assumed to have a magnitude of 70 kV/m, which is about a factor of 2 lower than the minimum field $E_{s}^+$ needed for positive streamer growth at 6.5 km altitude. Initially, new positive streamer links appear at random points due to the small-scale electric field enhancement mechanism described by Iudin et al.^1^. Each newly-formed streamer link connecting neighboring nodes with indexes *i* and *j* provides charge separation with the magnitudes of separated charges $\delta q_{i,j}=\alpha E_{i,j}$, where coefficient $\alpha$ is set (using trial-and-error approach) to $10^{-14}$ (C m)/V. All the newly-formed streamer links are oriented along the local electric field direction. Their collective effect leads to intense local electric field fluctuations. These fluctuations, in turn, create conditions in which the streamer growth (beyond the distance between the neighboring nodes) becomes possible.

In this model, creation of a streamer link is a stochastic process, the probability of which depends on the magnitude of the electric field between two neighboring nodes with indexes *i* and *j* and is given by the following formula:

7 $$\begin{aligned} P(E_{i,j})=1-\exp\left\{ -\left( \frac{E_{i,j}}{E_{s}^\pm }\right) ^{2.5}\right\} , \end{aligned}$$

where $E_{s}^+=$150 kV/m at 6.5 km and $E_{s}^-=2E_{s}^+$ are the electric fields needed for the positive and negative streamer propagation, respectively. Directions of growth of positive and negative streamers are determined by the direction of the local electric field. As a streamer system grows and polarizes, its conductivity increases (potentially reaching the values corresponding to “hot” channels), which serves to push the electric field toward the discharge-tree periphery.

Initial conductivity $\sigma _0$ of a newly-formed streamer link, which can be viewed as a multitude of branched and interacting streamers^63,64^, is set to $\sigma _0=10^{-5}\,\mathrm {S}/\mathrm {m}$. In the course of link evolution, its conductivity $\sigma$ increases and decreases via Joule heating and dissipation effects (cooling), respectively, which are all described, following Syssoev et al.^63,64^, by the following equation:

8 $$\begin{aligned} \frac{\partial \sigma }{\partial t} = (\eta E^2 - \beta )\sigma , \end{aligned}$$

where $\eta =6\cdot 10^{-5}$ $\text {m}^2$/($\text {V}^2$ s) and $\beta =6\cdot 10^3$ $\text {s}^{-1}$ are the parameters which represent the rates of link heating and cooling, respectively.

The electric field along a link is found as the potential difference between the nodes at its ends divided by the length of the link. This field gradually relaxes from the pre-breakdown value to the hot-channel value under the action of potential equalization by currents flowing through all the discharge tree channels. For each link joining two nodes with indexes *i* and *j*, this current is found from Ohm’s law as

9 $$\begin{aligned} I_{i,j} = \sigma _{i,j} \pi r^2 E_{i,j}, \end{aligned}$$

where *r* is the equivalent radius of the link (channel segment) which is set to 2 mm, regardless of its conductivity (actually, the cross-sectional area of links in the model is determined by the 1-m grid spacing, as explained in detail by Iudin et al.^30^).

The model time step at which the discharge-tree structure changes is 7.1 μs, while all the evolutionary equations are solved with a much smaller time step of 3.5 ns.

In this model, positive streamers gradually develop into bipolar streamer systems, whose trunks and major branches can eventually become hot (see channel segments shown in red in Fig. 4) and can interconnect (see the rightmost snapshot at $t=283$ μs, where the hot channel segments of two discharge trees with $\Delta V=3$ MV and $\Delta V=1.4$ MV are about to come in contact).

## Data Availability

Video file visualizing evolution of 3D streamer/hot-channel system, three snapshots of which are shown in Fig. 4, is available online at 10.5281/zenodo.4015943^65^. Additional data that support the findings of this study are available from the corresponding author (email: iudin@ipfran.ru) upon request.

## Abbreviations

AGP: Above the grounded plane
CRS: Cosmic ray shower
EAS: Extensive air shower (same as CRS)
EICRs: Elevated ionic conductivity regions
FPB: Fast positive breakdown
HCS: Hot channel segments embedded in streamer networks
IPCs: Ion production centers
RICSs: Residual ion concentration spots
RREA: Relativistic runaway electron avalanche
UPFs: Unusual plasma formations
